# Watt matters most – Survey data results of private passenger vehicle owners and commercial vehicle drivers

**DOI:** 10.1016/j.dib.2023.109942

**Published:** 2023-12-14

**Authors:** Maximilian Zähringer, Teresa Junior, Lennart Adenaw

**Affiliations:** Technical University of Munich, TUM School of Engineering and Design, Chair of Automotive Technology, Boltzmannstr. 15, 85748 Garching, Germany

**Keywords:** Charging behavior, Battery electric vehicle, Survey Data

## Abstract

This data set contains the questionnaires and respective responses from two joint surveys dealing with the actual and anticipated charging behaviors of battery electric vehicle (BEV) drivers and professional truck drivers. During a period of 2 months in early 2023, a total of 348 responses were collected using an online survey built on the LimeSurvey framework. Participants were recruited using a database of professional truck drivers available to the authors and an open recruitment process based on QR codes attached to charging stations in the Munich area. In the fast-paced electromobility market, this data set constitutes one of the few sources of insights into the charging behaviors of current and future users of electrified vehicles. It may be used to calibrate behavioral models or derive design decisions for future charging infrastructures.

Specifications Table

**Table:** 

Subject	Automotive Engineering
Specific subject area	Survey, Charging behavior, Truck electrification, BEV, Charging infrastructure design
Type of data	Survey Questionnaire Survey Responses
How the data were acquired	The data were acquired by two online questionnaires which were conducted over a period of two months in 2023. The questionnaires were implemented using the LimeSurvey framework and formed the basis of two different surveys: a truck and a BEV survey. The BEV survey yielded a total of 300 responses, while the truck survey was answered by 48 persons. Both questionnaires contain questions regarding socio-demographics, vehicle electrification, driving and charging behavior as well as attitudes toward aspects of electromobility.
Data format	Raw data Preprocessed
Description of data collection	Two online surveys, one for BEV, and one for truck drivers, were set up and yielded a total of 348 responses which are all included in this data set. For the BEV survey, stickers with QR codes linking to the respective online survey were attached to 56 charging stations in Munich and its surroundings. The truck survey was sent to professional truck drivers known to the Chair of Automotive Technology of the Technical University of Munich.
Data source location	Institution: Technical University of Munich, TUM School of Engineering and Design, Chair of Automotive Technology City/Town/Region: D-85748 Garching Country: Germany Data acquisition: Germany Longitude (BEV survey – charging points) between 11.402° E and 15.478° E Latitude (BEV survey – charging points) between 47.082° N and 48.555° N
Data accessibility	Repository name: CBS – Charging Behavior Survey Data identification number: 10.5281/zenodo.10083066 Direct URL to data: https://zenodo.org/records/10083066 Instructions for accessing these data: The repository contains a set of compressed csv files. A concise description of their contents is provided in this research article.

## 1 Value of the Data

• Researchers, automotive engineers, planners of charging infrastructure, and public authorities may benefit from the data set as it provides a basis for the user-oriented design of charging infrastructure for both passenger cars and battery electric trucks (BET). Based on this data set, credible user journeys may be deduced and serve as a basis for requirements engineering. Furthermore, behavioral models for the charging behavior of BEV users may be calibrated using the data provided herein.
• Planners of highway charging infrastructure for battery electric trucks may find the joint survey useful in transferring knowledge from the more mature BEV market onto the BET market.
• The data set presented is therefore helpful for the current expansion of existing charging infrastructure for BEV and for the rollout of charging hubs for BET and, thus, addresses energy suppliers, charge point operator (CPO) and policy makers.
• Despite its small sample size in comparison to the total amount of BEV on the road, this data set is a valuable addition to the state of the art of charging behavior analysis and modeling. This is because few other surveys on the matter exist. Additionally, the electromobility market saw considerable growth in the early 2020s, leading to the constant necessity of insights into its status. Such insights are provided by our sample qualitatively and – to a limited extent – quantitatively.
• We present two sets of data from two surveys, one for BEV drivers and one for truck drivers. While the data set of the BEV surveys with a sample size of 300 (197 fully completed) can be classified as high, the data set of the truck survey with a sample size of 48 (36 fully completed) can be classified as lower regarding representativeness. It should be seen as a first indication.

## 2 Objective

The objectives of the two joint surveys presented herein are twofold: First, the surveys are designed to shed light on the current charging behavior of BEV users. Second, a baseline for the understanding of the choice of highway service areas and charging stations for the use case of battery electric trucks is to be established. The truck survey is designed to deliver starting points for the development of a future transportation management system (TMS) for heavy electric commercial vehicles. The One reason for two surveys is that truck drivers currently do not have any experience with electromobility and therefore the charging behavior of BEV drivers is taken as a basis for identifying future charging needs in the commercial vehicle sector as realistically as possible. This makes it possible to transfer the potential for improvement identified by the survey of BEV drivers to the charging infrastructure for commercial vehicles that will be built in the future. Similarities and differences between the main factors in charging station choices of BEV users and potential ones of BET drivers can be derived. Another reason for the two surveys is that cars and trucks cover a substantial proportion of vehicles dependent on public charging infrastructure. In particular, public service areas with appropriate charging infrastructure must be aware of and meet the needs of both groups.

The primary objectives represent only a portion of the information found in the data. The following description of the data collected should be considered in the context of these objectives. Additional insights can be gleaned from the data sets provided.

## 3 Data Description

In this chapter, we first discuss the data provided and its form before evaluating the data from the BEV and truck survey using descriptive statistics. Finally, we look at individual aspects relating to the charging behavior of BEV drivers and the resting behavior of truck drivers.

### 3.1 Data availability and data format

The data repository available at https://zenodo.org/records/10046440 contains three data file types, all in CSV format for ease of import to multiple data science tools:

1. The surveys’ complete results are provided in both German (original) and English (translated) language (*bev_survey_ger.csv, bev_survey_eng.csv, truck_survey_ger.csv, truck_survey_eng.csv*).
2. Question encodings are given by *bev_question_encoding.csv* and *truck_question_encoding.csv*. These encoding files contain the original question texts, their English translations, the corresponding column name mapping to column names in the survey data CSV, and the data type per question.
3. *bev_response_translation.csv* and *truck_response_translation.csv* comprise all original response options and their English translations.

Together, these files ensure an easy reconstruction of the original survey, its responses in German, an English translation, and practical usability of the data within typical data analysis frameworks. In addition to the data set in CSV format, we provide two Python scripts to import the data files as data frames. This ensures the ease and fast use of the data.

The following sections provide further insights into the main files that make up the data set.

### 3.2 Main data files: {bev/truck}_survey_{ger/eng}.csv

These four files contain all questions from the two surveys (BEV/truck) as columns and the sets of responses per participant as rows. Instead of stating the original question texts as column names, all column names are replaced by a unique encoding given by a variable name that is human-readable, hints toward the original question text, and complies with typical variable naming conventions. Additionally, metadata describing each response set is included (response id, last survey page visited by the respondent, initial language setting of the device used to fill in the survey). Table 1 summarizes the contents of the main data files.

**Table 1:** Components, data types, and content description of the main data files.

Table Component	Data Type	Description
Headers (first line)	String	Survey questions or response metadata identifiable by encoding variable names
Rows	Various (primarily categorical variables)	Coherent sets of responses per participant. Datatypes for each column are given by the respective encoding CSV.

The English and German data sets contain the same column name encodings. These two CSV files only differ concerning the response language, with German being the original.

### 3.3 Question encodings: {bev/truck}_question_encoding.csv

The question encoding files contain the original survey questions in German, their English translations, a mapping to their column names in the main data files, and a definition of the data types of the responses. Table 2 provides a specification of the columns of these files:

**Table 2:** Columns and content descriptions of the question encoding CSV files.

Column	Description
question_text_ger	German question text as used in the original survey.
question_text_eng	English translation for the German question text (added during post-processing).
column_name	Variable name used as column name to encode the question text in the main data sets (*{bev/truck}_survey_{ger/eng}.csv*)
column_dtype	Definition of the datatype of the responses in the main data sets. The data sets contain the following data types: • Boolean variables: ‘boolean’ • Categorical variables: ‘category’ • 64-bit Integers: ‘Int64’

### 3.4 Response translations: {bev/truck}_response_translation.csv

The response translation files contain 1:1 translations of the available German responses to English. These translations were added during post-processing and were unavailable to the respondents during the survey, which was exclusively conducted in German. Since boolean and integer columns within the main data sets do not need translation, only translations of categorical variables (see encoding files) are given. Table 3 contains a definition of the columns of these files:

**Table 3:** Columns and content descriptions of the response translation CSV files

Column	Description
category_ger	German response category option as used in the original survey.
category_eng	English translation for the German response text (added during post-processing).

### 3.5 Classification of collected data: demographic analysis

The demographic analysis of the collected survey data is shown for both the BEV driver and the truck driver datasets. Based on this, we give a brief classification of the representativeness of the participant groups by comparison with other available data in this context.

*Private battery electric vehicle drivers*

**Fig. 1:**
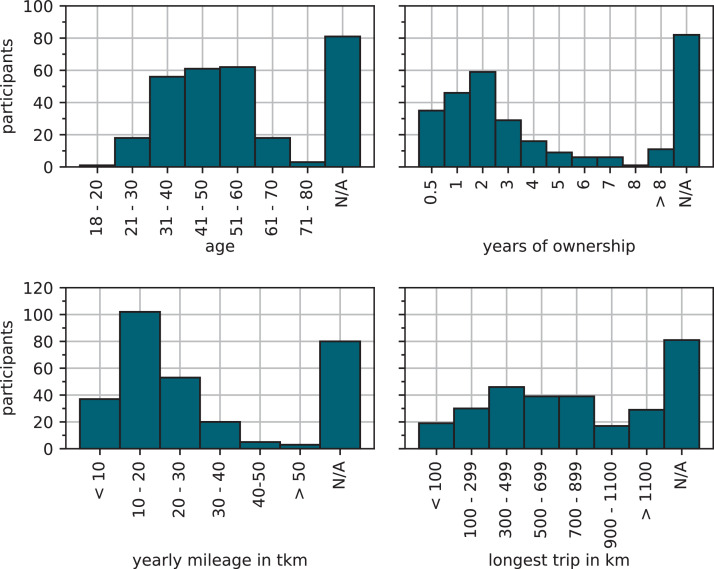
Descriptive analysis of demographic and mobility-related data for participant group of BEV survey.

The survey for BEV drivers was answered by $N_{tot}=300$ participants, of which $N_{com}=197$ answered the survey completely. The participants are divided into $N_{f}=20$ women, $N_{m}=163$ men, und $N_{d}=1$ diverse people (N/A=3). Fig. 1 shows the distribution for age, years of BEV ownership, annual mileage and longest distance travelled by the participant group.

**Fig. 2:**
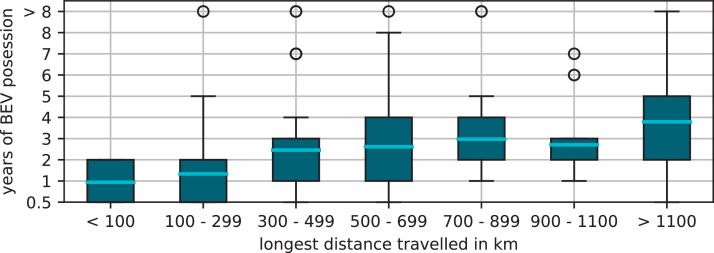
Correlation of years of ownership with longest distance travelled. A small trend of an increase of the longest trip traveled with a BEV by an increase of years of possession is observable.

Most of the participants are between 31 and 60 years old and owned their BEV for less than 3 years. The majority of participants drives 10,000 and 20,000 km per year, which is in line with the average annual mileage of 13,323 km of passenger car drivers in Germany [1]. The longest trips conducted by the greater part of the BEV drivers are between 300 and 900km long. In comparison with a comprehensive survey from 2016 by Pessier et al. [2] we find an increase in the longest distance traveled. The years of BEV ownership by participants shows good consistency with the historical market ramp-up of BEV in Germany [3]. Fig. 2 shows an analysis of the dependency between the years of BEV ownership and the longest distance traveled. We can observe that as the number of years of BEV ownership grows the longest travel distance also escalates, reaching up to 700 km within 2 to 3years. Longer Journeys appear to exhibit no discernible dependence on the duration of BEV ownership. While the mobility-related data of the above analysis (Fig. 1) is in good agreement with other surveys and statistical data from Germany, the data set published here shows a strong gender bias. 85% of the fully completed surveys were answered by men. Especially for the data related to charging behavior in Section (3.3) this should be considered. We provide an analogous insight into the dataset of truck drivers survey next.

*Commercial truck drivers*

**Fig. 3:**
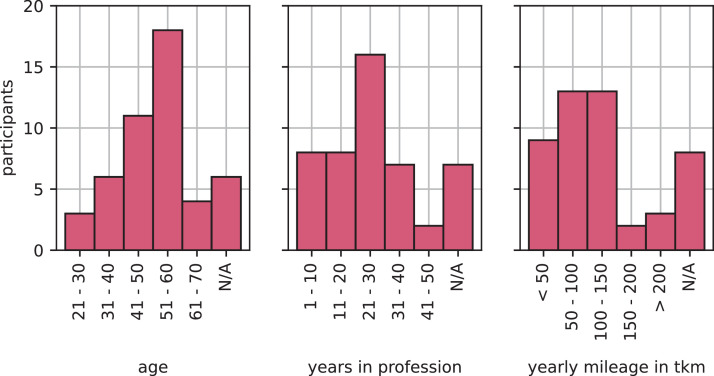
Descriptive analysis of demographic and driving-related data for participant group of truck drivers survey.

The survey for truck drivers was answered by $N_{tot}=48$ participants, of which $N_{com}=36$ answered the survey completely. The participants are divided into $N_{f}=3$ women, $N_{m}=32$ men, und $N_{d}=1$ diverse people. Fig. 3 shows the distributions of age, years in the profession and annual mileage of the survey participants.

Almost half (44.4%) of the surveyed truck are between 51 and 60 years old and more than half have been doing their job for more than 20 years. Approximately equivalent segments divide the annual milage, ranging from less than 50 tkm to 150 tkm. Only a few participants stated that they drive more than 150 tkm per year. This results align well with the annual mileage of heavy-duty vehicles according to the Kraftfahrt-Bundesamt [1] which reports an average annual mileage of 34,034 km for commercial vehicles over 7.5 t permissible total weight and of 89,714 km for tractor units. Furthermore, 52% of the participants state that they drive primarily in distribution transport and about 40% in long-distance transport. To categorize the findings in (Chapter 3.4), it is imperative to note that 80% of the participants have never driven a BET, and 72% express a lack of interest in rapid change. Among the drivers with BET experience, the question about a quick change was answered in a mixed way. This is in alignment with the study conducted by Aral in 2023 [4]. With a total number of participants of 36, the sample size can be considered small.

Since the population of the sample is unknow, and consequently an estimation of the standard deviation is not possible, an exact minimum sample size can only be roughly estimated. For a confidence interval of 0.9, an error of 0.1 and a standard deviation of 0.5, a sample size of about 100 participants would be required [5]. For even more accuracy, a higher number of samples is required. The gender bias observed in BEV drivers is also observable here. In the next section, we first show charging-related insights for BEV drivers.

### 3.6 Charging behavior of private battery electric vehicle owners

**Fig. 4:**
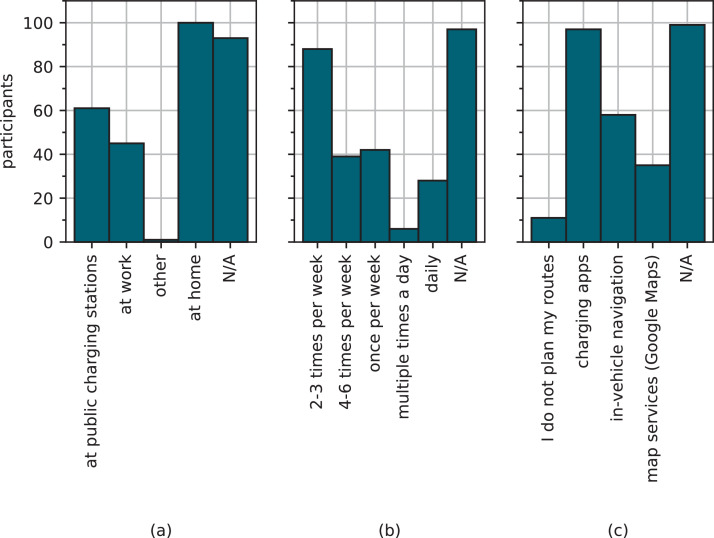
Descriptive analysis of charging behavior of BEV drivers. (a) Shows the most common charging locations, which is at home, followed by public charging stations. (b) Outlines the number of charging events per week, which most often is 2-3 times. (c) Shows the distribution of the chosen method for planning long-distance trips with BEV. The most popular method is to use specific charging apps like *A Better Route Planner*[6].

Fig. 4 provides insights into the charging behavior of the BEV survey participants. About half of the participants indicated their home as the most frequently used place for charging their vehicle. The other half of the participants use public charging facilities or charging facilities at work in roughly equal proportions (Fig. 4 (a)). The analysis of the data set further shows that more than 80% of the participants charge their vehicle at least 2-3 times per week, with 2-3 times per week being the most frequently indicated bin (Fig. 4 (b)).

**Fig. 5:**
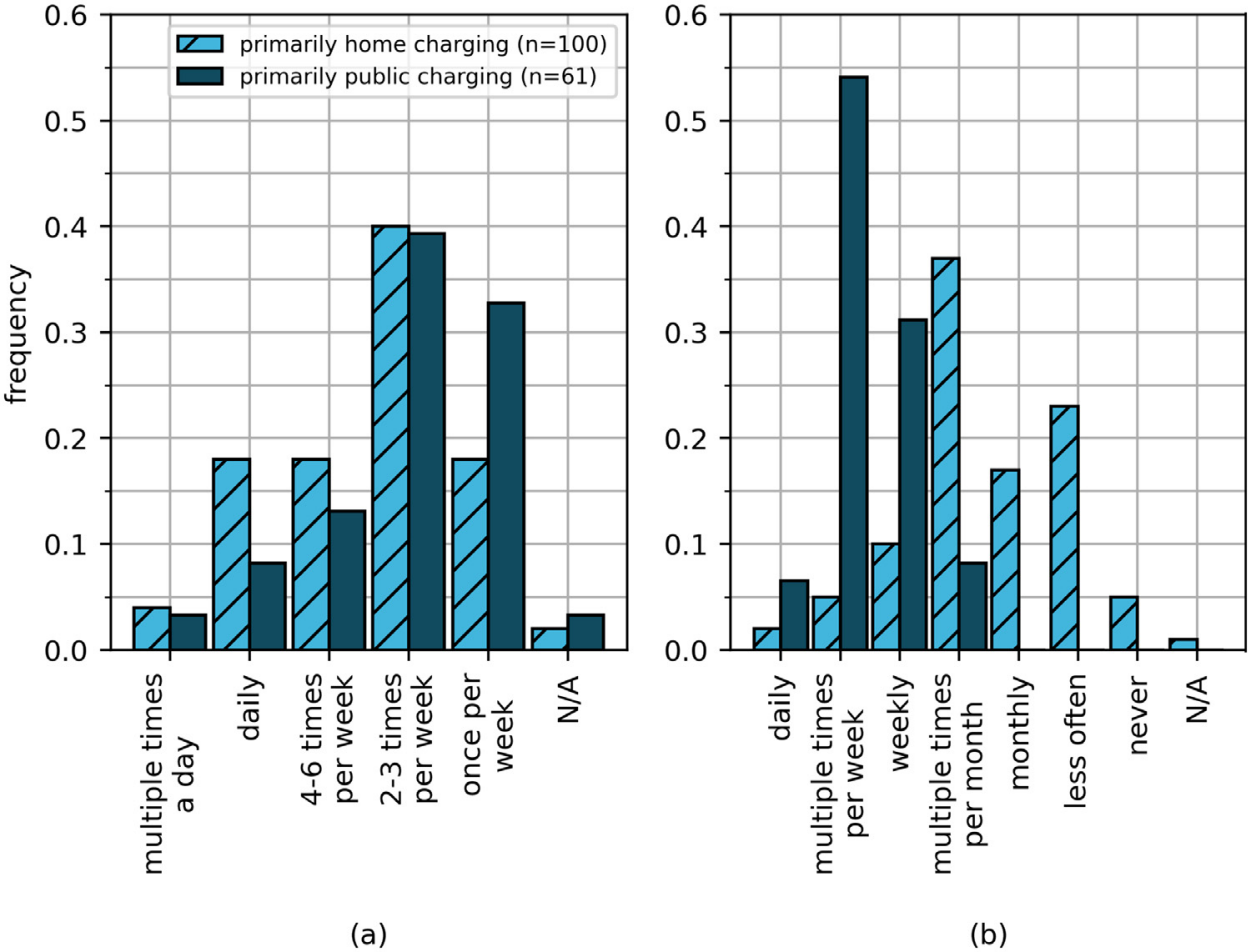
(a): Respondents’ overall charging frequency depending on their primary charging location, (b): Respondents’ public charging frequency depending on their primary charging location.

Private charger availability is expected to influence charging behavior due to the ease with which users can engage in charging activities (no searching for parking spaces, exclusive availability), less expensive energy costs at one's place of living in contrast to revenue-based public charging infrastructure, and increased comfort (less walking, no time connection thresholds). Thus, when analyzing the data sets provided, it is important to consider private charger availability at home and workplaces. The survey asked the participants to reveal their primary charging location (“What is the most common place to charge your electric vehicle?”). The largest sub-group of the respondents reported that they primarily charge at home (Fig. 4 (b)), which is in line with other surveys that indicate home charging opportunities are widely available [7]. The second largest group of respondents states that they mainly charge in public places. Fig. 5 (a) and (b) show the dependence of charging behavior on private home charger availability as given by the survey. Fig. 5 (a) compares the overall charging frequency (“How often do you charge your electric vehicle on average per week?”) for respondents who primarily charge at home and those who primarily charge in public places. Within this survey, people with a home charging availability charge more often than people who mostly utilize public options.

Fig. 5 (b) depicts the distribution of public charging frequencies for the two groups (“How often do you charge your electric vehicle at a public charging station?”). It shows that survey participants who mainly use public chargers also utilize public charging options significantly more often than those who primarily charge at home. Building on the insights from Fig. 5, the place of primary charging can be considered an important explanatory variable that ought to be considered when working with the provided data sets.

For routes with a length exceeding the vehicle range, BEV users are confronted with selecting suitable charging options on the road. In addition to the vehicle own systems, well-known navigation services such as Google Maps or specific applications such as *A Better Route Planner* [6] can also be used to search for charging points. The survey results show that the specific charging apps are used most frequently, followed by the vehicle's own navigation systems (Fig. 4 (c)). Several factors can influence the specific selection of a charging point. In addition to technical or economic influences such as charging power and charging costs, subjective factors can also be decisive. Therefore, we asked the participants to rank the importance of different criteria when choosing a charging station.

**Fig. 6:**
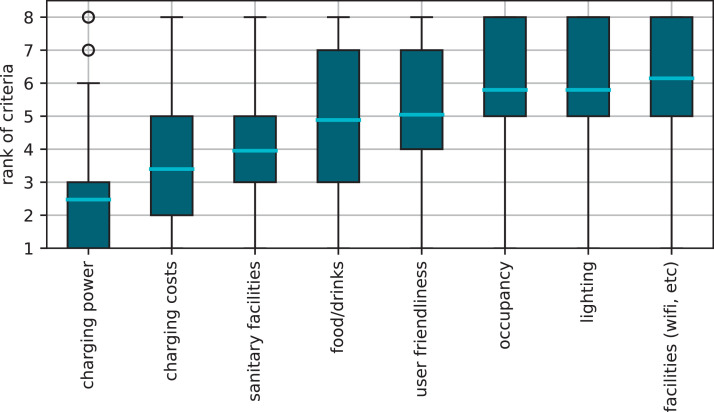
Ranking according to the importance of the considered criteria when choosing a public (fast) charging station. The most important criteria are charging power and costs as well as clean sanitary facilities. Not-answered counts are not considered here.

Fig. 6 shows that charging power and charging costs are the dominant factors. However, it is shown that clean sanitary facilities are equally important in some cases, although the average rank is lower. It should be noted, that the published data set contains further analysis of questions relating to BEV charging, which are not shown here.

So far, we have investigated the charging behavior and the choice of public charging points for private BEV users. For battery electric trucks (BET) an area-wide charging infrastructure does not yet exist. A charging infrastructure tailored to the user, i.e. truck drivers, can increase the attractiveness of individual charging sites. In the next paragraph, we therefore provide an insight into todays criteria taken into account, when choosing rest sites. From this, recommendations for action can be derived for the design of dedicated truck charging spots.

### 3.7 Service site selection of commercial truck drivers

**Fig. 7:**
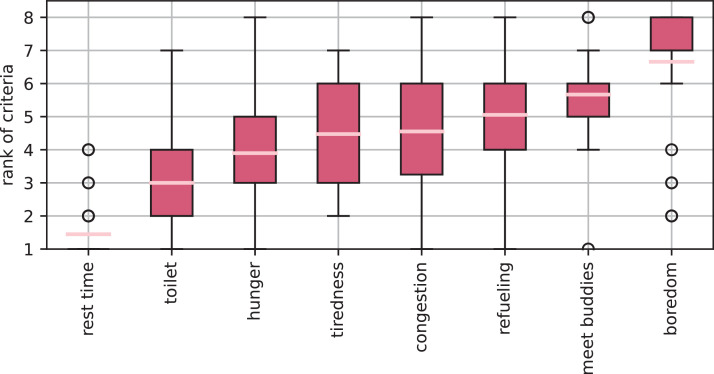
Ranking of reasons that lead to truck drivers taking breaks. The primary reason for stopping is the mandatory rest time. Beside this, the most popular reasons are toilet needs and hunger.

Due to the mandatory driving and rest periods, truck drivers are forced to take breaks both during the day and overnight. Especially in long-distance transport application, these breaks take place at public rest areas. In order to understand where and when truck drivers take their breaks, we posed two separate questions regarding the participants’ break preferences. First, the truck drivers were asked to rank the reasons for taking a break in the first place. Second, we asked them to rank potential influencing factors for the choice of the specific rest area by importance. Fig. 7 shows an evaluation of the importance of individual reasons for driving to a rest site. As expected, the legally required rest break is the main reason. The two most frequently cited reasons are the need for a toilet and hunger. Reasons such as fatigue, traffic jams or refueling are rated as less important.

**Fig. 8:**
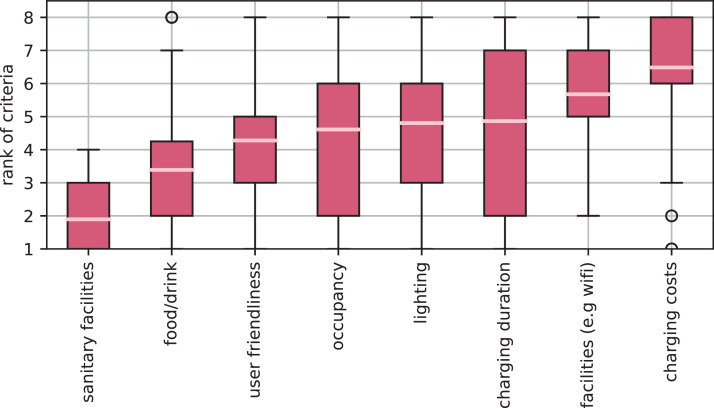
Ranking according to the importance of the considered criteria when choosing a service site. In alignment with Fig. 6 clean sanitary facilities and the food offer are the high-ranked criteria when choosing a service site.

For the construction of future charging parks for BET, both existing resting facilities, so called brownfield areas, and new locations (greenfield) can be considered. The needs of customers can be taken into account, especially in the case of new facilities. For this purpose, Fig. 8 shows which criteria are currently taken into consideration when choosing rest facilities. Sanitary facilities were named as the most important criterion, followed by the food on offer. Criteria of medium importance are the occupancy rate or the general user-friendliness. The respondents were also asked to imagine driving a battery-electric truck that also needs to be recharged during rest breaks. This shows that charging time was rated significantly more important than charging costs, what can be attributed to the high time pressure drivers are operating under [8]. In summary, the provision of sanitary facilities and an attractive food offer are the key features of a rest area and should be considered in the construction of future charging sites for BET in contrast to current charging sites for passenger vehicles.

Understanding user needs is essential for the acceptance of electromobility and, thus, a necessary precursor for climate-neutral individual mobility. In the preceding paragraphs, we have analyzed an excerpt of the collected data, which allows us to make recommendations for the design of current charging options for BEV as well as for future charging locations for battery electric commercial vehicles. The published data set provides further information in a processed form. With regard to BEV, for example, questions about charging speed and time savings due to shorter charging processes were also examined. The fully questionnaires of the both surveys are included in the appendix.

## 4 Experimental Design, Materials and Methods

Finally, we take a brief look at the survey design and the acquisition of participants.

### 4.1 Survey design

The sample size of the BEV driver survey, consisting of nearly 200 fully completed surveys, coupled with the participants acquisition method described in the subsequent paragraph, allows to infer that the data set of the BEV driver survey can be considered as representative. However, due to its sample size of 36 fully completed surveys, the data set of the truck driver survey is limited in its representativeness. The validity of both surveys was assessed in terms of content validity by conducting a pretest with two experienced researches in this area. As part of the pretest, besides content-related aspects such as the clarity of the questions, the ability to answer the questionnaire on various end devices and the time required were also tested.

We rate the reliability of the BEV driver survey as high because the results align with comparable questions in another survey [2]. However, we did not perform a retest. It is difficult to assess the reliability of the truck survey as there are less comparable surveys, and their results are not publicly available. However, the here presented data set can serve as a basis for further surveys.

Furthermore, short, comprehensible, and unambiguous formulations of the questions ensure truthful answers. The surveys consist of a combination of closed and open questions, both to ensure uniform data collection and to gather additional information. The BEV driver survey is divided into four parts and contains 22 questions. The truck driver survey is split into two parts and contains 23 questions. The question concerning the ranking of influencing factors according to their relevance in choosing a rest/charging station is the only mandatory question in both surveys. Answering the remaining questions is voluntary and can be skipped. Both surveys take about 5 to 10 minutes to complete.

### 4.2 Survey accessibility and data acquisition

Both surveys were made accessible via a QR-Code or direct internet link using the well-known professional software *LimeSurvey* [9]. Care was taken to ensure that both surveys could also be completed using mobile devices. We ensure this by processing a pretest of the survey with several different devices and numerous participants.

**Fig. 9:**
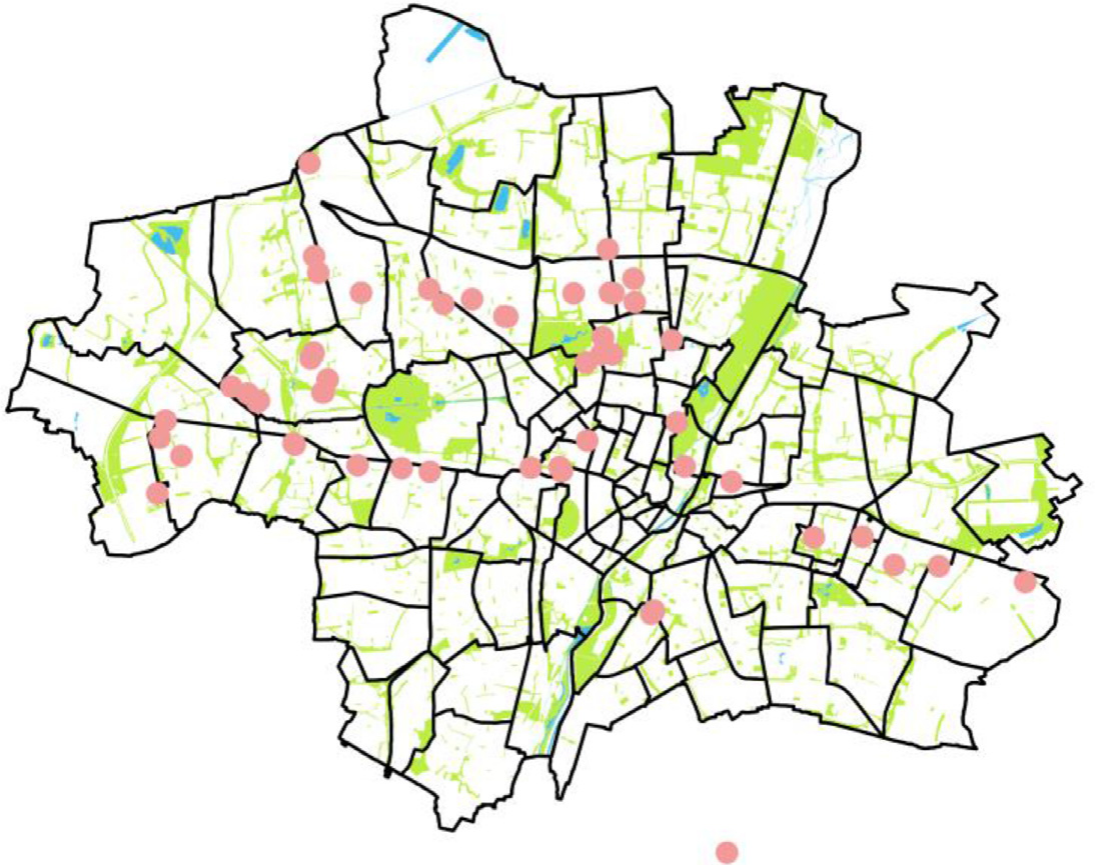
The red dots show the position of charging stations equipped with survey accessibility sticker for BEV survey in Munich, Germany. The green areas show existing green fields in the urban area of Munich, Germany as a reference. The stickers were distributed in a way that ensures spatial coverage and the inclusion of different location types (e,g. urban, suburban).

We used several channels to acquire participants. For the BEV survey, the QR codes (A.1) were stuck on public charging points in the Munich urban area in order to directly acquire participants in the relevant charging situations. Fig. 9 below shows the position of the stickered charging sites.

The participants of the truck driver survey were recruited via an internal database of the Technical University of Munich which contains contact data of interested truck drivers. The list of contact information was generated through a public call for truck drivers interested in research topics. Furthermore, participants were acquired in truck-specific online forums and facebook groups. Incentives for participation were not used in either survey.

## Limitations

The dataset of the BEV drivers survey, with 197 complete responses, shows a significantly higher representativeness than the dataset of the truck drivers survey, with only 36 complete answers. It should be noted here that a clear gender bias was observed in both surveys (Ch. 3.5). Both, the representativeness and the bias should be considered when analyzing and interpreting the datasets. Further, on the one hand, BEV drivers and research-savvy truck drivers were explicitly addressed by both surveys. Therefore, both samples do not represent the social spectrum of the population in the country where data was collected.

## Ethics Statements

The study was conducted according to the guidelines of the Declaration of Helsinki [10]. Ethical review and approval were waived for this study as no ethical issues were involved (e.g., no vulnerable populations, no collection of sensitive issues, no distressing situations, invasive activities or collection of biological materials).

## CRediT authorship contribution statement

**Maximilian Zähringer:** Conceptualization, Methodology, Formal analysis, Funding acquisition, Investigation, Data curation, Writing – original draft, Writing – review & editing. **Teresa Junior:** Conceptualization, Methodology, Formal analysis, Funding acquisition, Investigation, Data curation, Writing – original draft. **Lennart Adenaw:** Investigation, Data curation, Visualization, Writing – original draft, Writing – review & editing, Supervision.

## Figures and Tables

**Fig. A.1:**
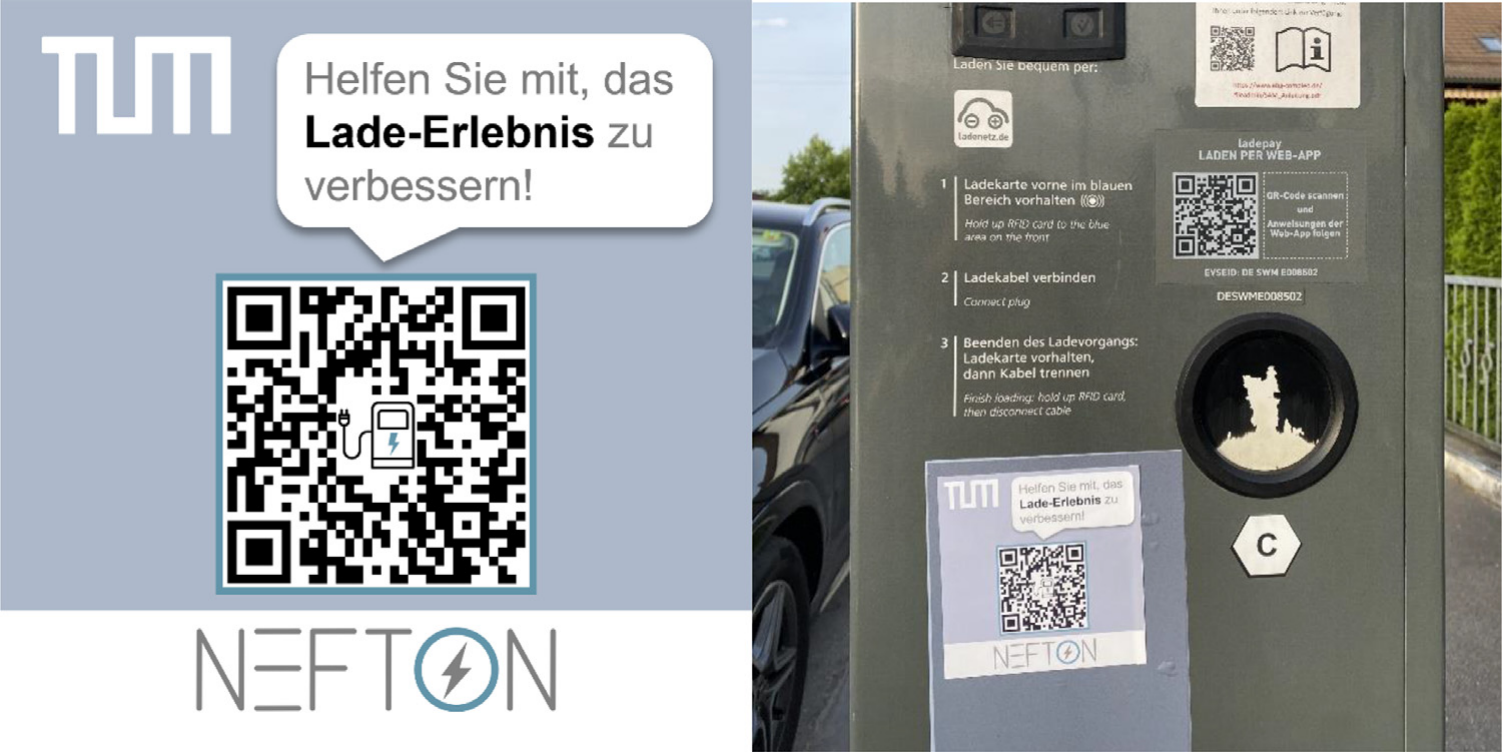
Used stickers with access to the survey for battery electric vehicle drivers. The stickers are positioned near the payment panel or inlet for the charging cable. All public charging stations in Fig. 10 used these stickers.

## Data Availability

CBS - Charging Behavior Survey (Original data) (Zenodo)

## Appendix

### A.2 Translated survey for battery electric vehicle drivers

• *What gender do you identify as?*

[] Female
[] Male
[] Diverse

• *What is your age?*

[] 18 – 20
[] 21 – 30
[] 31 – 40
[] 41 – 50
[] 51 – 60
[] 61 – 70
[] 71 – 80

• *For how many years do you own your electric vehicle?*

[] 0,5
[] 1
[] 2
[] 3
[] 4
[] 5
[] 6
[] 7
[] 8
[] > 8

• *How many kilometers do you drive on average per year with your electric vehicle?*

[] < 10000
[] 10000 - 19999
[] 20000 - 29999
[] 30000 - 39999
[] 40000 - 50000
[] > 50000

• *How often do you charge your electric vehicle on average per week?*

[] Multiple times a day
[] Daily
[] 4 – 6 times per week
[] 2 – 3 times per week
[] Once per week

• *What is the most common place to charge your electric vehicle?*

[] At home
[] At work
[] At public charging stations
[] Others:

• *How often do you charge your electric vehicle at a public charging station?*

[] Daily
[] Multiple times per week
[] Weekly
[] Multiple times per month
[] Monthly
[] Less often
[] Never

• *How satisfied are you with the expansion of the public charging infrastructure?*

[] Very satisfied
[] Rather satisfied
[] Neutral
[] Rather dissatisfied
[] Very dissatisfied

• *How satisfied are you with the reliability of the public charging infrastructure?*

[] Very satisfied
[] Rather satisfied
[] Neutral
[] Rather dissatisfied
[] Very dissatisfied

• *How often do you charge at highway charging parks/rest areas?*

[] Daily
[] Multiple times per week
[] Weekly
[] Multiple times per month
[] Monthly
[] Multiple times a year
[] Less often
[] Never

• *Are you interested in increasing the charging speed of your vehicle?*

[] Yes
[] No

• *When choosing a public charging station, do you make sure that "green" electricity is available?*

[] Yes
[] No
[] Other:

• *How do you plan long distance trips on unfamiliar routes with at least one charge?*

[] In-vehicle navigation
[] Charging apps
[] Map services (Google Maps)
[] I do not plan my routes.

• *Additional charging stops might lead to time savings on longer trips. Which time saving would be necessary to make you use additional charging stops?*

*For example: Two stops with 10 minutes charging each instead of one stop with 30 minutes charging = 10 minutes time saving*
[] 5 min
[] 10 min
[] 15 min
[] 20 min
[] 25 min
[] I would never accept another charging stop.
*Your vehicle predicts a SOC of 80% for a charging time of 30 minutes. After 30 minutes your SOC is only at 70%.*

• *How annoyed are you about the wrong prediction?*

[] Very annoyed
[] Rather annoyed
[] Neutral
[] Rather not annoyed
[] Not annoyed at all

• *Would you wait for reaching a SOC of 80% or would you continue driving with a lower SOC of 70%?*

[] Waiting
[] Driving

• *You are looking for a charging station during a long distance trip: What are the most important criteria for your choice of a charging station? (Please select all, even if unimportant)*

*Please number each box in order of preference, starting with 1 through 8.*
[] Sanitary facilities
[] Food/Drinks
[] Charging power
[] Facilities (wifi, signage, leisure facilities, etc.)
[] Lighting
[] Cost of charging
[] User friendliness
[] Occupancy

• *Do you include further criteria in your decision?*

[] Yes
[] No

• *What are the criteria?*
• *Are there any situations that would change your classification of the criteria above? (stress, hunger, ...)*

[] Yes
[] No

• *What are the situations?*

### A.3 Translated survey for truck drivers

• *What gender do you identify as?*

[] Female
[] Male
[] Diverse

• *What is your age?*

[] 18 – 20
[] 21 – 30
[] 31 – 40
[] 41 – 50
[] 51 – 60
[] 61 – 70
[] 71 – 80

• *For how many years are you a professional truck driver?*

[] 1 – 10
[] 11 – 20
[] 21 – 30
[] 31 – 40
[] 41 – 50
[] > 50

• *How many kilometers do you drive on average per year?*

[] < 50,000
[] 50,000 – 100,000
[] 100,000 – 150,000
[] 150,000 – 200,000
[] > 200,000

• *Do you drive more in distribution or long haul traffic?*

[] Distribution
[] Long haul

• *Do you drive for several days at a stretch on a regular basis (overnight)?*

[] Yes
[] No

• *How many days per week do you drive on average?*

[] 1
[] 2
[] 3
[] 4
[] 5
[] 6
[] 7

• *Have you ever driven an electric truck?*

[] Yes
[] No

• *Would you appreciate a quick switch to a battery electric truck in daily use?*

[] Yes
[] No

• *If so, why?*
• *If so, why?*
• *How long are your breaks within your shift on average?*

[] < 30 min
[] 30 min
[] 45 min
[] 60 min
[] 90 min
[] > 90 min

• *Do you use the break split (45 min divided into 15 min and 30 min)?*

[] Yes
[] No

• *You are driving your electric truck, which needs to be charged. The charging process can take different lengths of time at different charging stations. Would you accept an additional charging stop to save time on your trip?*

[] Yes
[] No

• *Which navigation system do you use?*

[] In-vehicle navigation
[] Fleet management navigation
[] Smartphone navigation app
[] No navigation

• *What is the most common reason you take a break?*

[] Rest time
[] Refueling
[] Boredom
[] Hunger
[] Toilet needs
[] Congestion
[] Meeting with driving buddies
[] Tiredness

• *You are looking for a rest area: What are the most important criteria for your choice?*

[] Sanitary facilities
[] Food/drinks
[] Short charging duration
[] Facilities (wifi, signage, leisure facilities, etc.)
[] Lighting
[] Cost of charging
[] User friendliness
[] Occupancy

• *Do you include further criteria in your decision?*

[] Yes
[] No

• *What are the criteria?*
• *Do any of the following factors change your ranking criteria for your choice of a rest area?*

[] Time pressure
[] Congestion
[] Low cost budget
[] Tiredness
[] Hunger
[] Day/Night
[] Nothing applies.

• *How satisfied are you overall with resting areas in Germany?*

[] Very satisfied
[] Rather satisfied
[] Neutral
[] Rather dissatisfied
[] Very dissatisfied

• *What bothers you most about resting areas in Germany?*
• *What do you like best about resting areas in Germany?*
